# Non-Invasive Stem Cell Therapy in a Rat Model for Retinal Degeneration and Vascular Pathology

**DOI:** 10.1371/journal.pone.0009200

**Published:** 2010-02-15

**Authors:** Shaomei Wang, Bin Lu, Sergei Girman, Jie Duan, Trevor McFarland, Qing-shuo Zhang, Markus Grompe, Grazyna Adamus, Binoy Appukuttan, Raymond Lund

**Affiliations:** 1 Casey Eye Institute, Oregon Health & Science University, Portland, Oregon, United States of America; 2 Oregon Stem Cell Center, Oregon Health & Science University, Portland, Oregon, United States of America; Tufts University, United States of America

## Abstract

**Background:**

Retinitis pigmentosa (RP) is characterized by progressive night blindness, visual field loss, altered vascular permeability and loss of central vision. Currently there is no effective treatment available except gene replacement therapy has shown promise in a few patients with specific gene defects. There is an urgent need to develop therapies that offer generic neuro-and vascular-protective effects with non-invasive intervention. Here we explored the potential of systemic administration of pluripotent bone marrow-derived mesenchymal stem cells (MSCs) to rescue vision and associated vascular pathology in the Royal College Surgeons (RCS) rat, a well-established animal model for RP.

**Methodology/Principal Findings:**

Animals received syngeneic MSCs (1×10^6^ cells) by tail vein at an age before major photoreceptor loss. Principal results: both rod and cone photoreceptors were preserved (5–6 cells thick) at the time when control animal has a single layer of photoreceptors remained; Visual function was significantly preserved compared with controls as determined by visual acuity and luminance threshold recording from the superior colliculus; The number of pathological vascular complexes (abnormal vessels associated with migrating pigment epithelium cells) and area of vascular leakage that would ordinarily develop were dramatically reduced; Semi-quantitative RT-PCR analysis indicated there was upregulation of growth factors and immunohistochemistry revealed that there was an increase in neurotrophic factors within eyes of animals that received MSCs.

**Conclusions/Significance:**

These results underscore the potential application of MSCs in treating retinal degeneration. The advantages of this non-invasive cell-based therapy are: cells are easily isolated and can be expanded in large quantity for autologous graft; hypoimmunogenic nature as allogeneic donors; less controversial in nature than other stem cells; can be readministered with minor discomfort. Therefore, MSCs may prove to be the ideal cell source for auto-cell therapy for retinal degeneration and other ocular vascular diseases.

## Introduction

Retinitis pigmentosa (RP) refers to a subset of inherited retinal degenerations, for which over 180 disease associated loci have been mapped and of these over 130 genes have been identified that when mutated result in severe vision impairment. An estimated 100,000 people in the U.S. have RP [1], [2], [3], [4], with a worldwide prevalence between 1 in 3000 to 1 in 7000. Retinitis pigmentosa is not only genetically heterogeneous but can be inherited in multiple forms, including autosomal dominant, autosomal recessive, X-linked, nonsyndromic and digenic-diallelic. A universal feature of all forms of RP is initial degeneration of photoreceptors and with time the pathology involves the inner retina, leading to a loss in its lamination, vascular leakage, invasion of RPE cells into the retina and subsequent loss of ganglion cells [5], [6], [7]. The majority of people with RP are usually legally blind by age 40–50, with visual symptoms manifesting in the early teens. Notably, there is no effective treatment available. Experimental animal models that mimic the human RP condition allow investigation and development of potential treatments. Viral mediated delivery of a normal copy of the affected gene has lead to partial reversal of the phenotypic changes in animal models and has led to human clinical trials [8], [9], [10], [11], [12], [13]. However, specific genetic defects have been found in only a few of the known retinal degenerative diseases, which thereby limit the potential application of gene therapy to those few patients. A generic blanket therapy for all retinal dystrophies may be a better global strategy, and indeed therapies with calcium channel blockers, vitamin supplementation and neuroprotective growth factors have been tested although in some cases with limited success [2], [14], [15], [16]. Cell-based therapy, especially the development of stem cell biology for application in treating neurodegenerative diseases to the retina has been shown to be effective. Direct injection of donor cells into vitreous does not have much merit, as donor cells tend to cover the back of the lens and block the passage of light into the eye, and thus preventing functional tests to determine efficacy of treatment. Intravitreal implantation of encapsulated factor secreting cells has the disadvantage of non-specific exposure of intraocular structures to potentially deleterious levels of growth factor, and there is limitation of allowing repeat implantation, which is required to sustain long-term efficacy. An advantage of delivering donor cells via subretinal injection method is that the therapeutic material is placed directly in the space where the defective RPE cells or degenerating photoreceptors are targeted, and in this fashion both photoreceptors and visual function can be partially preserved [17], [18], [19]. However, donor cells are usually distributed across at most about a quarter of the total retinal area, the rest of the retina undergoes progressive degeneration, especially the development of the secondary vascular pathology, which compromises donor cell survival and related beneficial effect. An attractive therapeutic intervention would be one that affords generic neuro-and vascular-protective effects via a non-invasive method and bestows protection to both rod and cone photoreceptors. The pluripotent bone marrow-derived mesenchymal stem cells (MSCs) are an ideal cell source for therapy of inherited or degenerative disease, because of its autologous characteristic, ease of isolation; secreting growth factors know to be neuro-vascular protective, less contentious relative to other stem cells [20], [21], [22], [23]. We found that MSCs preserved vision and limited vascular pathology when intravenously injected into the Royal College Surgeon (RCS) rat, a well-established animal model of RP.

A mutation in the gene for the receptor tyrosine kinase Mertk, in the RCS rat, results in dysfunction of retinal pigment epithelium (RPE) cells [24]. Compromising the ability of the RPE to phagocytize photoreceptor outer segments leads to a progressive loss of both rods and cone cells overtime in the RCS rat [25], [26]. Interestingly, mutation within the human orthologue of Mertk results in RP, whereby patients exhibit progressive poor visual acuity and visual field losses with age [27].

## Results

### Neuroprotection of Cones and Rods

**Figure 1:**
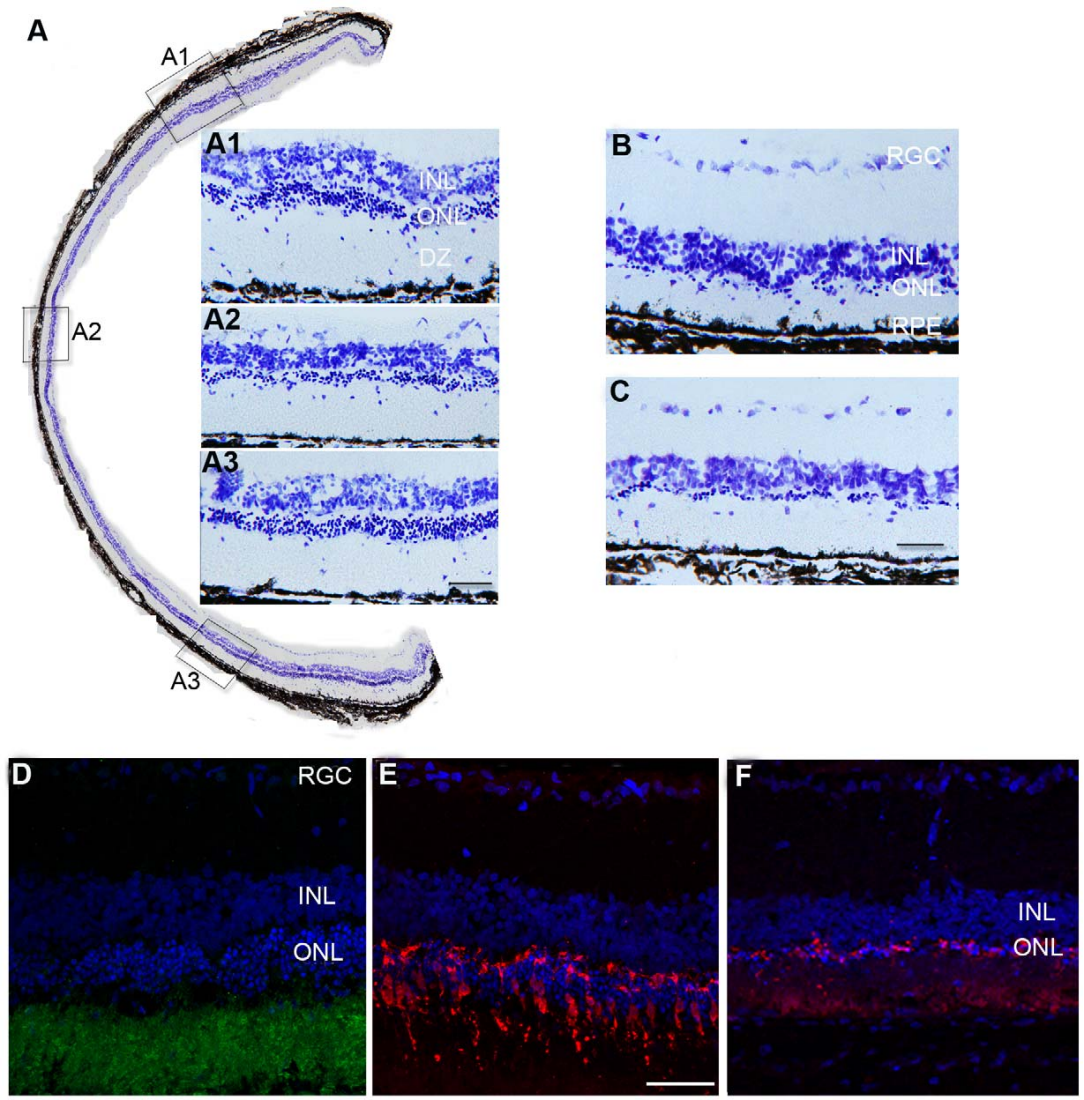
Rod and cone protection. A. Retinal sections stained with cresyl violet indicate substantial preservation of photoreceptors across the retina in MSC treated eyes at P90, while in control eyes (sham injected (B) and untreated (C)): only a single layer of photoreceptors remained. A1, A2 &A3: higher power images showing preservation of photoreceptors from the insets A1, A2 &A3 in A. D, E&F: confocal images showing rhodopsin (green in D) and cone arrestin (red in E) positive staining at P90 in MSC treated retina, while in sham injected retina, cone arrestin staining was dramatically reduced (F). All sections were counterstained with DAPI (blue) (scale bars equal 50 µm).

In the RCS rat by postnatal day (P) 90 only a single layer of photoreceptors remains compared to the 10 layers observed at P30. To investigate whether MSCs could provide a neuroprotective effect, we isolated and injected intravenously syngeneic MSCs into RCS rats at P30 (n = 12), at which time the retinal degeneration is at an early stage. Eyes were collected and processed at P90 to determine efficacy of MSC treatment in comparison to controls (sham injection (carrying medium alone): n = 8, and untreated: n = 8). Retinal sections were stained with cresyl violet for examining general retinal lamination and with photoreceptor cell-specific antibodies (rhodopsin, cone arrestin), which showed the preservation of cone and rod photoreceptors within the MSC treated animals. We found that photoreceptors were substantially rescued across the retina (Figure 1A). Although an uneven distribution of cell layer thickness was noted with more prominent rescue in peripheral than central retina. There were 5–6 layers of photoreceptors in the peripheral retina compared with 2–3 layers in the central retina (Figure 1: A1&A3 vs. A2). The retina appeared orderly laminated. In contrast, there was a single layer of photoreceptor remaining in both sham injected (Figure 1B) and untreated retinas (Figure 1C). Morphologically both rod and cone photoreceptors were rescued (Figure 1 D&E). Further analysis revealed that cone density was 18±3 cells/300 µm in retina that had received MSCs (20±2 cells/300 µm in wild type); while in control retina (Figure 1F) it was impossible to conduct meaningful counts due to severe degeneration.

### Functional Preservation of Photoreceptors

**Figure 2:**
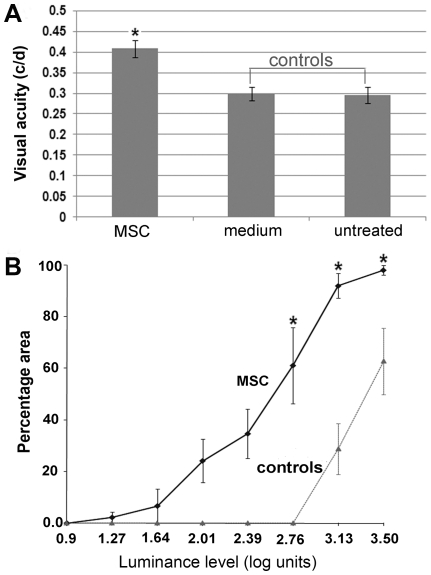
Preservation of visual function. A. Visual acuity tested by Optomotor response. Unrestrained animals were placed on a platform, where they tracked the grating with reflexive head movements. The acuity threshold was quantified by increasing the spatial frequency of the grating. RCS rats received MSCs and medium injection via tail vein at P30 and tested at P90. Visual acuity was significantly better in MSC treated eyes compared with controls (P<0.001). A value of 0.43 c/d was recorded, which was 78% of normal value (0.55 c/d in wild-type). B. The luminance threshold was evaluated by recording single and multiunit activity close to the surface of the superior colliculus (SC). It measures functional sensitivity across the visual field, which in turn provides a topographic indication of the magnitude and area of photoreceptor rescue across the retina. MSC treated rats recorded around P90–100 revealed significantly lower threshold than controls (P<0.005), for example, over 60% of the SC area had threshold at 2.76 log units in MSC treated eyes, no detectable response in control eyes.

In the RCS rat, visual function deteriorates as photoreceptors are lost. Visual acuity in the RCS rat as tested by an optokinetic system (under photopic condition) has been shown to decrease with age from 0.5 cycle/degree (c/d) at P30 to 0.3 c/d at P90 [28]. This test is non-invasive, rapid and allows for repeated measurements of spatial frequency and contrast sensitivity thresholds of the optokinetic response (OKR). Another test for functional photoreceptors is a luminance threshold (LT) recording from the superior colliculus (SC) under standard background luminance level. In the RCS rat, the LT was elevated from 1.2 log units at P30 to 3 log units at P90 [29](<0.4 log units in wild rat). Although LT recording is time-consuming, it measures functional sensitivity across the visual field, which in turn provides a topographic indication of the magnitude and area of photoreceptor rescue across the whole retina. To examine whether MSCs preserved visual function after intravenous administration, we conducted the aforementioned functional tests that correlated very well with the morphological neuroprotective data. The OKR analysis revealed that there was significant difference between MSC treated and control eyes (P<0.001) (Figure 2A). An average of 0.41±0.01 c/d was recorded at P90 in MSC treated animals (n = 12), whereas 0.30±0.01 c/d in medium injected (n = 8) and 0.29±0.02 c/d in untreated (n = 8) controls were observed. Luminance threshold recordings revealed that MSC injected eyes (n = 6) produced thresholds less than 2.76 log units over 60% of the total SC area; while in controls (n = 6), no SC area produced thresholds less than 2.76 log units. Thus, MSC treated eyes had significantly lower threshold than untreated eyes (p<0.05), indicating a convincing degree of functional preservation (Figure 2B).

### Vascular Protection

**Figure 3:**
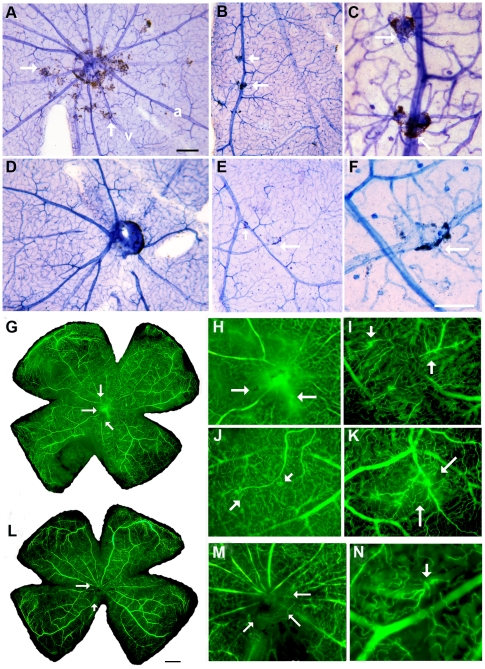
Vascular protection. A–F: Retinal whole mount was stained with NADPH-diaphorase: A. typical vascular pathology in the eye at P90 in untreated RCS rat: vascular complexes (abnormal vessels associated with RPE cells) were mainly located around the optic nerve disc (arrows) and spread peripheral with age. B. vascular complexes in the middle to peripheral retina (arrows). C. high power image showing vascular complexes (arrows) from B. D. RCS retina treated with MSCs at P90: the vascular complexes were dramatically reduced around the optic nerve disc. E. two vascular complexes (arrows) in the middle field of the retina. F. high power image from E showing vascular complexes (arrow). G–L. animal was perfused with FITC-dextran, whole mount was prepared: G. typical vascular leakage, mainly around the optic disc in untreated eye at P90. H–K. high power images from G showing vascular leakage (arrows in H) and abnormal vessels (arrows in I–K). L. MSC treated retina, the vascular leakage around the optic nerve disc was greatly reduced. M&N. high power images from L showing much reduced leakage (arrows in M) and small abnormal vessels (arrow in N) (Scale bars equal 250 µm for A, D, G &L; 100 µm for F).

In the RCS rat, retinal vascular pathology develops as photoreceptors degenerate. Leakage from vessels within the deep capillary plexus is first detected at P60, using horseradish peroxidase (HRP) perfusion method, and this seepage is initially located around the optic nerve disc and eventually with increasing age spreads to the whole retina [30]. In the RCS rat vascular complexes, determined as abnormal vessels associated with clusters of retinal pigment epithelium cells (RPE), are clearly evident by P90. The RPE cells appear to migrate along the abnormal vessels and form pathological vascular complexes. To examine whether MSCs confer a vascular-protective role, retinal vessels were stained with the nicotinamide adenine dinucleotide phosphate-diaphorase (NADPH-diaphorase) on whole mount preparation as described previously [30]. The NADPH-diaphorase staining reveals the outline of retinal vessels and also allows identification of migrating RPE cells attached to pathological vessels. We found that the number of pathological vascular complexes was dramatically reduced in MSC treated retina (n = 8), compared with medium (n = 6) or untreated controls (n = 6) (0–8 vs. 25–30 of vascular complexes; p<0.001) (Figure 3A vs.D). In controls, the vascular complexes were located immediately ventral to the optic nerve head, with spreading to middle and eventually to the peripheral parts of the retina. It is common to find many vascular complexes at multiple sites along a major vessel (Figure 3B). High power image showed the vessels were twisted and covered by pigment granules (Figure 3C). We also noticed that the vascular complexes in MSC treated retinas appeared to be smaller (Figure 3E&F) and if present usually isolated instead of clustered as observed in the controls.

To further examine vascular integrity, animals were injected with fluorescein isothiocyanate (FITC)-dextran via the tail vein and the retinal whole mount was examined under fluorescence microscopy. We found that typical leakage around the optic nerve head was observed in control animals at P90 (n = 6) (Figure 3G&H); abnormal vascular profiles (dilated, torturous with evidence of leakage) were seen in the mid to peripheral retina (Figure 3I–K) and it was common to see multiple abnormal vascular profiles on one vessel. However, vascular leakage and abnormal vascular profiles were dramatically reduced in animals that had received MSCs (n = 6) (Figure 3L–N). The abnormal vascular profiles were much smaller, isolated and located around the optic nerve disc and rarely seen anywhere else in the retina.

### Trophic Factors

**Figure 4:**
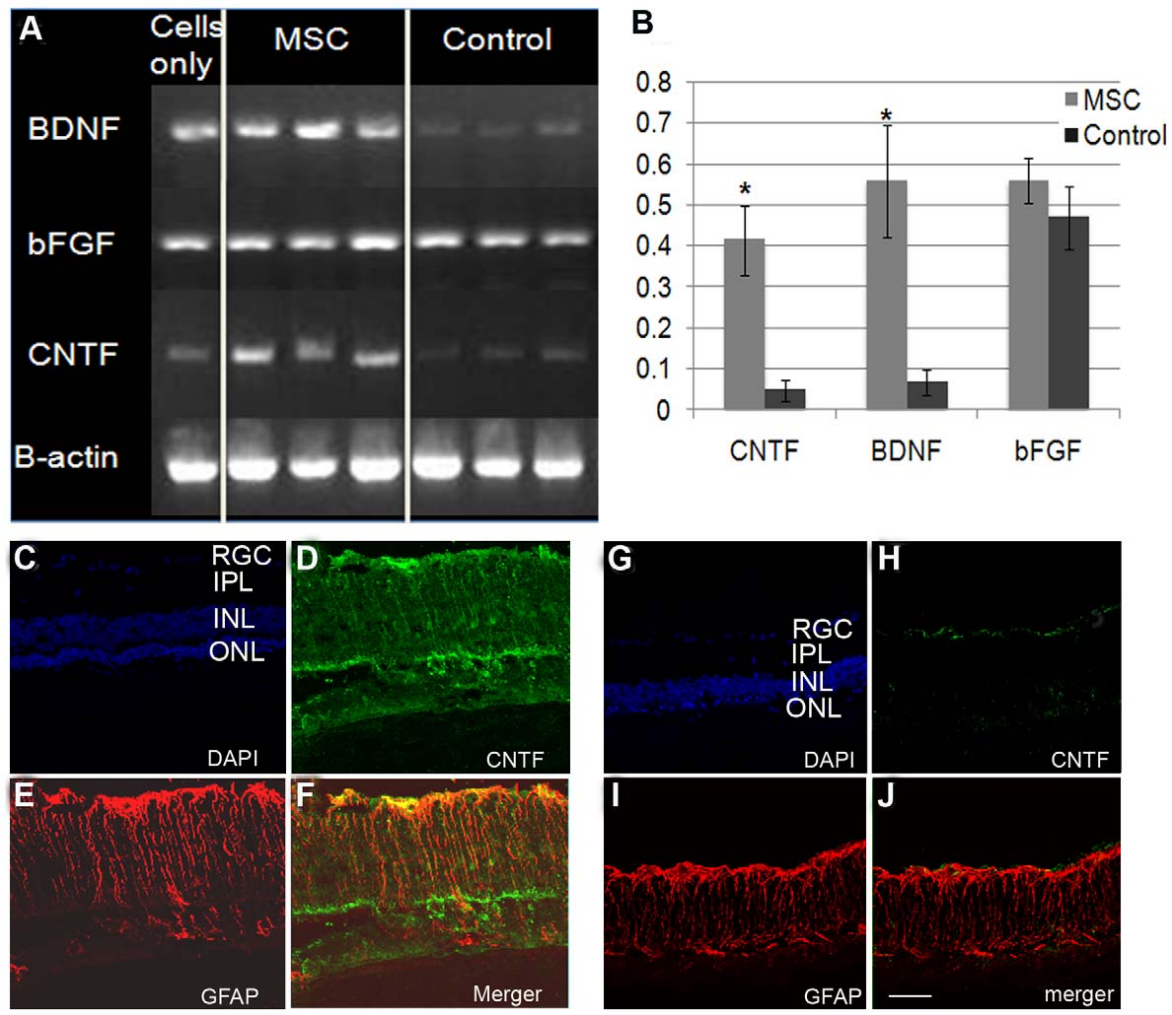
Upregulation of trophic factors. A. Semi-quantitative RT-PCR for CNTF, bFGF, BDNF and beta actin. Lane 1: RNA isolated from MSC prior to injection; Lane 2–4: RNA isolated from retinas treated with MSC; Lane 5–7: RNA isolated from non-treated control retinas. B. Densitometry analysis of CNTF, BDNF and bFGF in treated versus untreated samples. Beta actin was used to normalize the data for comparison. Level of CNTF and BDNF in the treated retinas were significantly higher than non-treated controls (p<0.05), while the level of bFGF in MSC treated retina did not increase significantly. C–J: confocal images of retinal sections double stained with antibodies to CNTF (green) and GFAP (red), counterstained with DAPI (blue in C and G) from MSC treated and controls. Strong CNTF staining in MSC treated retina (D) compared with untreated control (H); E&I: retinal sections stained with GFAP (red) showing upregulation of GFAP in Müller glia in both MSC treated and untreated control; F&J: merged images showing colocalization of CNTF and GFAP in MSC treated retina (F), which was not observed in untreated control (J) (Scale bar equals 50 µm).

We hypothesized that the neuro-vascular protection afforded by the introduction of MSCs was achieved by the increase in production of neurotrophic growth factors within the retina. To investigate this theory we performed semi-quantitative RT-PCR from retinal tissue isolated from animals at P90. We found that growth factors including ciliary neurotrophic factor (CNTF), basic fibroblast growth factor (bFGF), and brain derived neurotrhophic factor were upregulated in MSC treated eyes (n = 3) compared with control eyes (n = 3) (Figure 4A&B). However, only CNTF and BDNF were significantly increased over controls as determined by densitometry analysis. To determine the cells responsible for the increase in this growth factor production in the retina, antibodies against CNTF, bFGF and BDNF were applied to retinal sections of MSC-injected (Figure 4C) and control (Figure 4G). Strong staining of CNTF was found in MSC-injected retina compared with controls (Figure 4D vs.H). There were no obvious difference observed for the other proteins between MSC treated and controls (data not shown). The retinal sections were double stained with glial fibrillary acidic protein (GFAP) (Figure 4E&I) for Müller cells and CNTF, which revealed their co-localization in MSC-injected retina (Figure 4F), not in control (Figure 4J), suggesting that Müller glia cells upregulate expression of CNTF in response to the presence of MSC within the eye.

### Distribution of MSCs

**Figure 5:**
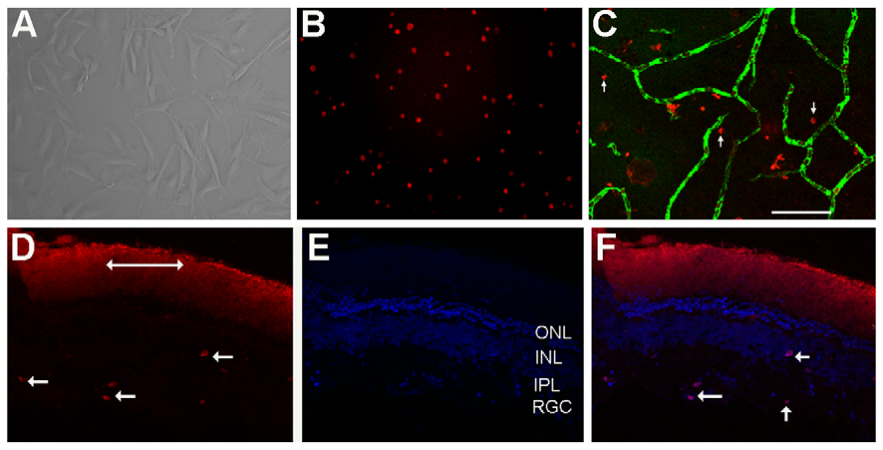
Distribution of MSCs. A. phase contrast microphotograph of bone marrow derived mesenchymal stem cells at passage 2. B. MSCs were preincubated with PKH26 before intravenous injection. C. PKH26 labeled MSCs in the retina two weeks after intravenous injection (arrows); blood vessels were perfused with FITC-dextran (green). D–F. showing PKH26 labeled MSCs in the retinal section (D, arrows pointing PKH26 labeled MSCs; double arrows indicating background staining in debris zone); sections counterstained with DAPI (E); F. merged image from D&E showing PKH26 labeled MSCs counterstained with DAPI (scale bar equals 100 µm).

To track the distribution of MSCs after systemic administration, MSCs (Figure 5A) were harvested and labeled with cell-linker PKH26 (Sigma) before injection (Figure 5B). Retinal whole mount and sections were examined 2 weeks after MSC injection. To see the relation between retinal vessels and MSCs, animals were perfused with FITC-dextran to highlight retinal vasculature before sacrifice. The PKH26 labeled MSCs were found in the eyes (Figure 5C, whole mount) and in other tissues including lungs, kidneys and liver (data not shown). In retinal sections, PKH labeled cells were seen in the retinal ganglion cells layers, inner and outer plexiform layers (Figure 5D–F).

This study demonstrated that bone marrow derived mesenchymal stem cells, when administrated intravenously, preserved both rod and cone photoreceptors, maintained visual function and limited vascular pathology.

## Discussion

Ocular vascular pathology is the most common cause of blindness and associated with several disorders. Age-related macular degeneration affects over 10 million individuals over age 65 in the United States alone. About 10−15% of these individuals suffer loss of vision as a direct effect of neovascularization of choroidal vessels [31], [32]. An estimated 100,000 people in the U.S. have RP with initial progressive photoreceptor loss followed by secondary vascular pathology (1). Over 40,000 patients with diabetes suffer from ocular compilations each year [33]. Many premature infants suffer from retinopathy of prematurity [34]. Vision loss has significant social and economic impact. This study demonstrates that renewable adult stem cells preserve vision and limit vascular pathology by systemic administration. Both rod and cone photoreceptors were preserved morphologically; visual functions tested by optomotor response and luminance threshold were significantly better than controls; vascular pathology including leakage and formation of vascular complexes was also dramatically reduced. The advantages of systemic administration of stem cells are that cells exert their effect over the whole retina, and multiple administrations can be easily performed if needed. The intravenous MSCs offer unique neuro-vascular protection as an auto-cell therapy.

Previous studies suggested that MSCs secrete a variety of cytokines and growth factors that have neuronal protective activities [20], [21], [35]. Our results indicated that the level of bFGF, BDNF and CNTF in MSC treated retina was higher than controls. Interestingly, CNTF was strongly expressed in Müller cells, which are the main source of trophic factors within the retina [36], [37]. The CNTF is a member of IL-6 family of cytokines that modulates survival of retinal neuronal cells. Up-regulation of endogenous CNTF is believed to promote photoreceptor survival, to protect them from mechanical injury. Direct intraocular injection of CNTF has been shown to retard photoreceptor death caused by inherited forms of retinal degeneration [38] or by light induced retinal damage [39]. A recent study by Hauk and colleagues [40] showed that intravitreal injection of toll-like receptor 2 agonist Pam3Cys (caused lens injury) can induce glial activation and upregulations of GFAP and CNTF, which significantly stimulated retinal ganglion cell axon regeneration into the injured optic nerve. An encapsulated cell therapy device that delivers CNTF has been used in clinical trials to rescue photoreceptors and is currently showing promising outcome [41]. Our current study showed that substantial photoreceptor rescue was observed across the whole retina and that both rods and cones were preserved. Systemic administration of whole bone marrow cells promoted photoreceptor survival in a mouse model of RP [42]. Sasahera and colleagues reported that the rescued photoreceptors were mainly cones in this RP model. In the current study we showed that both rod and cone photoreceptors were rescued at morphological and functional level. Further analysis indicated that cone density was comparable to the wild type rat; while in the RCS control retina, cone photoreceptors degenerated with a disorganized profile. It would appear that stem cells exert their effect over the whole retina when administered systemically. In comparison, subretinal delivery of cells including bone marrow derived cells [43], [44], [45], [46] usually results in rod and cone rescue close to the injection site and/or correlated with donor cell distribution, and thus there is no noticeable protection at sites distant from the grafted cells.

The vascular protection bestowed by MSCs is partly mediated by increased expression of angiopoietin-1/Tie2 and vascular endothelial growth factor/FLK1 in the animal model for stroke [47]. We performed RT-PCR and immunohistochemistry to test the presence of angiopoietin-1/Tie2 and vascular endothelial growth factor/FLK1 on retinal tissue isolated from MSC treated and untreated RCS rats. We observed no difference in the expression profiles between the control and treated animals (data not shown). It is possible that the mechanism of MSC mediated vascular protection in this degeneration model is acting through an alternative pathway. Further study is under way to investigate this phenomenon in the RCS model.

The mechanism by which MSCs home into degenerating eyes is not fully understood. Studies have shown that stem cell migration and organ-specific homing are regulated by chemokines and their receptors. The expression of CXCR4 has been reported on embryonic stem cells [48], [49] and bone marrow derived stem cells [50], [51], [52], [53]. The specific CXCR4 ligand, stromal cell-derived factor-1 (SDF-1) is expressed by several tissues and upregulated by injury or ischemia. The SDF-1/CXCR4 axis plays an important role in the recruitment of circulating progenitor cells to home to sites of ischemic injury to facilitate repair [54]. Our study showed that MSCs were found in the retina two weeks after intravenous injection. It is likely that retinal degeneration leads to upregulations of certain chemokines, which promote MSCs to home into the eye. It would be interesting to investigate whether CXCR4 and its ligand are involved; how the MSCs are distributed and differentiated in the eye with time; when is the best time to administrate MSCs to achieve optimal efficacy.

The results of this study provide preliminary evidence in support of potential clinical application, whereby a patient's own bone marrow cells can be used to treat retinal degeneration and ocular vascular pathology, such as that observed in diabetic retinopathy. Diabetic retinopathy is associated with increased capillary permeability, which can lead to retinal edema and retinal neovascularization. The MSCs can provide neuro-vascular protection and may avoid many of the unwanted potential side effects associated with the use of viral vectors in gene therapy. The issue of rejection associated with non-autologous stem cells may also be limited. The advantages of this non-invasive cell-based therapy are: cells are easily isolated and can be expanded in large quantity for autologous graft; hypoimmunogenic nature as allogeneic donors; less controversial in nature than other stem cells; can be readministered with minor discomfort and non surgical procedures. Currently, there are over 80 clinical trials using bone marrow derived cells to treat various human diseases. Therefore, MSCs may prove to be the ideal cell source for auto-cell therapy for retinal degeneration and other ocular vascular diseases.

## Methods

### Isolation and Culture of Rat MSCs

MSCs were obtained from the bone marrow of RCS rats (6–8 weeks old) according to the method previously described [55], [56]. Briefly, bone marrow was flushed from femurs and tibias with Dulbecco's modified Eagle's medium (DMEM)(Gibco, Invitrogen, USA) and centrifuged at 600 g for 10 minutes. Freshly isolated cells were resuspended in DMEM supplemented with 10% fetal bovine serum (HyClone, UT, USA), 100 U/ml penicillin G and 100 µg/ml streptomycin sulfate (Invitrogen, USA) and then seeded into T75 flasks (Corning, MA, USA). After 8 days, nonadherent cells were removed and adherent cells were detached with 0.05% trypsin/0.53 mM EDTA and replated. After 3 days, cells were detached with 0.1% trypsin/0.02% EDTA and plated at 2000 cells/cm^2^. MSCs were passaged upon reaching 50% confluency and cells from passage 2–4 were used for this study.

### Distribution of MSCs

To track the distribution of MSCs after systemic administration, cell-linker PKH26 (Sigma) was used according to manufacturer's protocol to label MSCs prior to injection. Retinal whole mount and sections were examined 2 weeks after MSC injection. To see the relation between retinal vessels and MSCs, animals were perfused with FITC-dextran to highlight retinal vasculature before sacrifice. Fluorescence microscopy was used to visualize PKH26 labeled MSCs within eyes and other tissues.

### Intravenous Administration of MSCs

MSC suspension containing 1 million cells/ml in balanced salt solution (BSS) was administered via tail vein using 31G needle to RCS rats at P30; as a control, age-matched RCS rats received BSS alone. These studies were conducted with approval and under the supervision of the Institutional Animal Care and Use Committee at the Oregon Health & Science University.

### Spatial Visual Acuity

Animals were tested for spatial visual acuity at P90 using an Optomotry testing apparatus (CerebraMechanics, Lethbridge, Can) [57]. The optomotry set-up comprises of four computer monitors arranged in a square that projected a virtual three-dimensional (3-D) space of a rotating cylinder lined with a vertical sine wave grating. Unrestrained animals were placed on a platform in the center of the square, where they tracked the grating with reflexive head movements. The spatial frequency of the grating was clamped at the viewing position by re-centering the ‘cylinder’ on the animal's head. The acuity threshold was quantified by increasing the spatial frequency of the grating using a psychophysics staircase progression until the following response was lost, thereby defining the acuity.

### Luminance Threshold

Luminance threshold (LT) was measured with the objective of providing parallel data to the surface of the superior colliculus (SC) using previously described procedures [29]. Recordings were made in rats using glass-coated tungsten electrodes (resistance: 0.5 MΩ; bandpass 500 Hz–5 KHz). The brightness of a 5° spot was varied using neutral density filters (minimum steps of 0.1 log unit) over a baseline level of visual acuity. The LT was evaluated by recording single and multiunit activity close to the 5.2 log units until a response double the background activity was obtained: this was defined as the threshold level for that point on the visual field. A total of 15–20 positions were recorded from each SC from P90–P95. Data was expressed as a graph of percentage of the SC area with a LT below defined levels and as raw results.

### Data Analysis

Statistical analyses were performed using GraphPad Prism version 5 for Windows (California, USA). All variables were expressed as mean± standard error of the mean. Differences between groups were compared by either Student's two tailed unpaired *t* test or analysis of variance. Newman-Keuls procedure was used for multiple comparision analysis. Differences were considered to be significant at *P*<0.05.

### Whole Mount Preparation

#### NADPH-diaphorase staining

Animals were perfused with PBS first, followed by 2% paraformaldehyde. The dorsal pole of each eye was marked before enucleation. Whole mount preparations of the retinas were prepared; four radial cuts were placed in the dorsal, ventral, temporal, and nasal poles permitting the retina to be laid flat. The retinas were postfixed for 30 minutes in the same fixative, washed and incubated in a solution containing 0.02% NADPH-diaphorase and 0.04% nitroblue tetrazolium (Sigma) in 3% Triton X-100 for 90 minutes at 37°C on a shaker. Retinas were washed with PBS, mounted on slides, dehydrated with alcohol and covered with DPX. Retinal whole mount was examined under a light microscope.

#### FITC-dextran perfusion

Animals were injected via tail vein with FITC-dextran and the dorsal pole of each eye was marked before enucleation. Eyes were fixed in 2% paraformaldehyde for 30 minutes, and then retinal whole mount was prepared as above and examined under a confocal microscope.

### Semi-Quantitative RT-PCR

RNA was isolated from cells and retinal tissue using the RNAqueous-4PCR kit (Ambion, USA) following the manufacturer's protocol including a DNAse I step. The RNA concentration for each sample was determined by UV spectrophotometry and quality was assessed by the ratio of 260/280. The iScript cDNA synthesis kit (Bio-Rad Laboratories, USA) was used to generate cDNA. Briefly, equal concentrations of RNA from each sample was reverse transcribed in the presence of 1X reaction buffer which included dNTPs, random hexamers, oligo(dT), MgCl_2_ and MMLV-derived reverse transcriptase. Samples were incubated for 5 minutes at 25°C, 30 minutes at 42°C and 5 minutes at 85°C. Following reverse transcription, PCR was performed using standard protocols for CNTF, bFGF, BDNF and β-actin. Briefly, 2 µl of each RT reaction were mixed with 23 µl of a PCR cocktail containing 1X PCR Buffer, 1.5 mM MgCl_2_, 1 unit of Taq polymerase, 10 mM dNTPS, 20 pmols of forward and reverse primers. Reactions were incubated at 95°C for 5 minutes followed by 30 cycles of 95°C for 30 seconds, 55°C for 30 seconds and 72°C for 30 seconds. PCR reactions were loaded onto 1% agarose gels containing ethidium bromide and visualized on a strategene UV gel-doc system. Digital photos of the results were used for densitometric analysis.

**CNTF** forward 5′-TGGGACAGTTGATTTAGGG-3′ and reverse primers 5′-GCTACATCTGCTTATCTTTGG-3′

**bFGF** forward 5′-GAGAAGAGCGACCCACAC-3′ and reverse primers 5′-GCAGACATTGGAAGAAACAG-3′

**BDNF** forward 5′-CCTGGCTGACACTTTTGAG-3′ and reverse primers 5′-ATTGGGTAGTTCGGCATTGCG-3′

**β-actin** forward 5′-GAGCGTGGCTACAGCTTCACCAC-3′ and reverse primers 5′-TACTCCTGCTTGCTGATCCACAT-3′

### Histology

#### Cresyl violet and immunohistochemistry

After all the functional tests, all animals were euthanized with an overdose of sodium pentobarbital (Sigma) and perfused with phosphate buffered saline (PBS). The eyes were then removed, immersed in 2% paraformaldehyde for one hour, infiltrated with sucrose, embedded in OCT and cut in sequence 10 µm horizontal sections apart on a cryostat. Every sixth section was placed on the same slide as the first section and a total of four sections (50 µm apart) were collected per slide. Approximately 80 slides were generated per eye, thus one eye contained a set of 16 and each set contained 5 slides. One slide from each set was stained with cresyl violet for assessing integrity of retinal lamination. The remaining slides were used for immunohistochemistry staining using retinal specific antibodies, following previously described protocols [43], and were examined by regular light and confocal microscopy. The retinal specific antibodies to rhodopsin (1∶1000, abcam, USA), cone arrestin (1∶3000, Chemicon), CNTF (1∶500, Santa Cruz, USA), GFAP (1∶1000, Sigma) and BDNF (1∶1000, Millipore) were used for immunohistochemistry.

## References

[1] Farrar GJ, Kenna PF, Humphries P (2002). On the genetics of retinitis pigmentosa and on mutation-independent approaches to therapeutic intervention.. EMBO J.

[2] Berson EL (1993). Retinitis pigmentosa. The Friedenwald Lecture.. Invest Ophthalmol Vis Sci.

[3] Hartong DT, Berson EL, Dryja TP (2006). Retinitis pigmentosa.. Lancet.

[4] Daiger SP, Bowne SJ, Sullivan LS (2007). Perspective on genes and mutations causing retinitis pigmentosa.. Arch Ophthalmol.

[5] Li ZY, Possin DE, Milam AH (1995). Histopathology of bone spicule pigmentation in retinitis pigmentosa.. Ophthalmology.

[6] Milam AH, Li ZY (1995). Retinal pathology in retinitis pigmentosa: Considerations for therapy..

[7] Santos A, Humayun MS, de Juan E, Greenburg RJ, Marsh MJ (1997). Preservation of the inner retina in retinitis pigmentosa. A morphometric analysis.. Arch Ophthalmol.

[8] Ali RR, Sarra GM, Stephens C, Alwis MD, Bainbridge JW (2000). Restoration of photoreceptor ultrastructure and function in retinal degeneration slow mice by gene therapy.. Nat Genet.

[9] Acland GM, Aguirre GD, Bennett J, Aleman TS, Cideciyan AV (2005). Long-term restoration of rod and cone vision by single dose rAAV-mediated gene transfer to the retina in a canine model of childhood blindness.. Mol Ther.

[10] Alexander JJ, Umino Y, Everhart D, Chang B, Min SH (2007). Restoration of cone vision in a mouse model of achromatopsia.. Nat Med.

[11] Bainbridge JW, Tan MH, Ali RR (2006). Gene therapy progress and prospects: the eye.. Gene Ther.

[12] Hauswirth WW, Aleman TS, Kaushal S, Cideciyan AV, Schwartz SB (2008). Treatment of leber congenital amaurosis due to RPE65 mutations by ocular subretinal injection of adeno-associated virus gene vector: short-term results of a phase I trial.. Hum Gene Ther.

[13] Williams DS (2008). Usher syndrome: animal models, retinal function of Usher proteins, and prospects for gene therapy.. Vision Res.

[14] Frasson M, Sahel JA, Fabre M, Simonutti M, Dreyfus H (1999). Retinitis pigmentosa: rod photoreceptor rescue by a calcium-channel blocker in the rd mouse.. Nat Med.

[15] Tao W, Wen R, Goddard MB, Sherman SD, O'Rourke PJ (2002). Encapsulated cell-based delivery of CNTF reduces photoreceptor degeneration in animal models of retinitis pigmentosa.. Invest Ophthalmol Vis Sci.

[16] Li T, Sandberg MA, Pawlyk BS, Rosner B, Hayes KC (1998). Effect of vitamin A supplementation on rhodopsin mutants threonine-17 --> methionine and proline-347 --> serine in transgenic mice and in cell cultures.. Proc Natl Acad Sci U S A.

[17] Lund RD, Adamson P, Sauve Y, Keegan DJ, Girman SV (2001). Subretinal transplantation of genetically modified human cell lines attenuates loss of visual function in dystrophic rats.. Proc Natl Acad Sci USA.

[18] Lu B, Malcuit C, Wang S, Girman S, Francis P (2009). Long-term Safety and Function of RPE from Human Embryonic Stem Cells in Preclinical Models of Macular Degeneration.. Stem Cells.

[19] Wang S, Girman S, Lu B, Bischoff N, Holmes T (2008). Long-term vision rescue by human neural progenitors in a rat model of photoreceptor degeneration.. Invest Ophthalmol Vis Sci.

[20] Caplan AI (2009). Why are MSCs therapeutic? New data: new insight.. J Pathol.

[21] Caplan AI, Dennis JE (2006). Mesenchymal stem cells as trophic mediators.. J Cell Biochem.

[22] Fukuda K (2003). Application of mesenchymal stem cells for the regeneration of cardiomyocyte and its use for cell transplantation therapy.. Hum Cell.

[23] Pittenger MF, Mackay AM, Beck SC, Jaiswal RK, Douglas R (1999). Multilineage potential of adult human mesenchymal stem cells.. Science.

[24] D'Cruz PM, Yasumura D, Weir J, Matthes MT, Abderrahim H (2000). Mutation of the receptor tyrosine kinase gene Mertk in the retinal dystrophic RCS rat.. Hum Mol Genet.

[25] Chaitin MH, Hall MO (1983). Defective ingestion of rod outer segments by cultured dystrophic rat pigment epithelial cells.. Invest Ophthalmol Vis Sci.

[26] Dowling JE, Sidman RL (1962). Inherited retinal dystrophy in the rat.. J Cell Biol.

[27] Gal A, Li Y, Thompson DA, Weir J, Orth U (2000). Mutations in MERTK, the human orthologue of the RCS rat retinal dystrophy gene, cause retinitis pigmentosa.. Nat Genet.

[28] Lund RD, Wang S, Klimanskaya I, Holmes T, Ramos-Kelsey R (2006). Human embryonic stem cell-derived cells rescue visual function in dystrophic RCS rats.. Cloning Stem Cells.

[29] Girman SV, Wang S, Lund RD (2005). Time course of deterioration of rod and cone function in RCS rat and the effects of subretinal cell grafting: a light- and dark-adaptation study.. Vision Res.

[30] Wang S, Villegas-Perez MP, Holmes T, Lawrence JM, Vidal-Sanz M (2003). Evolving neurovascular relationships in the RCS rat with age.. Curr Eye Res.

[31] Smith TC, Lee L (2007). Age related macular degeneration - new developments in treatment.. Aust Fam Physician.

[32] Dorrell M, Uusitalo-Jarvinen H, Aguilar E, Friedlander M (2007). Ocular neovascularization: basic mechanisms and therapeutic advances.. Surv Ophthalmol.

[33] Fong DS, Aiello L, Gardner TW, King GL, Blankenship G (2004). Retinopathy in diabetes.. Diabetes Care.

[34] Smith LE (2002). Pathogenesis of retinopathy of prematurity.. Acta Paediatr.

[35] Chen L, Tredget EE, Wu PY, Wu Y (2008). Paracrine factors of mesenchymal stem cells recruit macrophages and endothelial lineage cells and enhance wound healing.. PLoS ONE.

[36] Bringmann A, Pannicke T, Grosche J, Francke M, Wiedemann P (2006). Muller cells in the healthy and diseased retina.. Prog Retin Eye Res.

[37] Steinberg RH (1994). Survival factors in retinal degenerations.. Current Opinion in Neurobiology.

[38] Cayouette M, Behn D, Sendtner M, Lachapelle P, Gravel C (1998). Intraocular gene transfer of ciliary neurotrophic factor prevents death and increases responsiveness of rod photoreceptors in the retinal degeneration slow mouse.. J Neurosci.

[39] LaVail MM, Unoki K, Yasumura D, Matthes MT, Yancopoulos GD (1992). Multiple growth factors, cytokines, and neurotrophins rescue photoreceptors from the damaging effects of constant light.. Proc Natl Acad Sci U S A.

[40] Hauk TG, Leibinger M, Muller A, Andreadaki A, Knippschild U (2010). Stimulation of axon regeneration in the mature optic nerve by intravitreal application of the toll-like receptor 2 agonist Pam3Cys.. Invest Ophthalmol Vis Sci.

[41] MacDonald IM, Sauve Y, Sieving PA (2007). Preventing blindness in retinal disease: ciliary neurotrophic factor intraocular implants.. Can J Ophthalmol.

[42] Sasahara M, Otani A, Oishi A, Kojima H, Yodoi Y (2008). Activation of bone marrow-derived microglia promotes photoreceptor survival in inherited retinal degeneration.. Am J Pathol.

[43] Wang S, Lu B, Wood P, Lund RD (2005). Grafting of ARPE-19 and Schwann Cells to the Subretinal Space in RCS Rats.. Invest Ophthalmol Vis Sci.

[44] Wang S, Lu B, Lund RD (2005). Morphological changes in the Royal College of Surgeons rat retina during photoreceptor degeneration and after cell-based therapy.. J Comp Neurol.

[45] Inoue Y, Iriyama A, Ueno S, Takahashi H, Kondo M (2007). Subretinal transplantation of bone marrow mesenchymal stem cells delays retinal degeneration in the RCS rat model of retinal degeneration.. Exp Eye Res.

[46] Arnhold S, Heiduschka P, Klein H, Absenger Y, Basnaoglu S (2006). Adenovirally transduced bone marrow stromal cells differentiate into pigment epithelial cells and induce rescue effects in RCS rats.. Invest Ophthalmol Vis Sci.

[47] Zacharek A, Chen J, Cui X, Li A, Li Y (2007). Angiopoietin1/Tie2 and VEGF/Flk1 induced by MSC treatment amplifies angiogenesis and vascular stabilization after stroke.. J Cereb Blood Flow Metab.

[48] Guo Y, Graham-Evans B, Broxmeyer HE (2006). Murine embryonic stem cells secrete cytokines/growth modulators that enhance cell survival/anti-apoptosis and stimulate colony formation of murine hematopoietic progenitor cells.. Stem Cells.

[49] Guo Y, Hangoc G, Bian H, Pelus LM, Broxmeyer HE (2005). SDF-1/CXCL12 enhances survival and chemotaxis of murine embryonic stem cells and production of primitive and definitive hematopoietic progenitor cells.. Stem Cells.

[50] Hatch HM, Zheng D, Jorgensen ML, Petersen BE (2002). SDF-1alpha/CXCR4: a mechanism for hepatic oval cell activation and bone marrow stem cell recruitment to the injured liver of rats.. Cloning Stem Cells.

[51] Sordi V, Malosio ML, Marchesi F, Mercalli A, Melzi R (2005). Bone marrow mesenchymal stem cells express a restricted set of functionally active chemokine receptors capable of promoting migration to pancreatic islets.. Blood.

[52] Wynn RF, Hart CA, Corradi-Perini C, O'Neill L, Evans CA (2004). A small proportion of mesenchymal stem cells strongly expresses functionally active CXCR4 receptor capable of promoting migration to bone marrow.. Blood.

[53] Honczarenko M, Le Y, Swierkowski M, Ghiran I, Glodek AM (2006). Human bone marrow stromal cells express a distinct set of biologically functional chemokine receptors.. Stem Cells.

[54] Shi M, Li J, Liao L, Chen B, Li B (2007). Regulation of CXCR4 expression in human mesenchymal stem cells by cytokine treatment: role in homing efficiency in NOD/SCID mice.. Haematologica.

[55] Neuhuber B, Swanger SA, Howard L, Mackay A, Fischer I (2008). Effects of plating density and culture time on bone marrow stromal cell characteristics.. Exp Hematol.

[56] Lennon DP, Caplan AI (2006). I&solation of rat marrow-derived mesenchymal stem cells.. Exp Hematol.

[57] Prusky GT, Alam NM, Beekman S, Douglas RM (2004). Rapid quantification of adult and developing mouse spatial vision using a virtual optomotor system.. Invest Ophthalmol Vis Sci.

